# Special Section Guest Editorial: Molecular Neurophotonics

**DOI:** 10.1117/1.NPh.11.2.024200

**Published:** 2024-06-28

**Authors:** Ute Hochgeschwender, Robert E. Campbell, Hideaki Mizuno

**Affiliations:** a Central Michigan University, College of Medicine, Mount Pleasant, Michigan, United States; b The University of Tokyo, School of Science, Department of Chemistry, Tokyo, Japan; c Katholieke Universiteit (KU) Leuven, Biochemistry, Molecular, and Structural Biology Unit, Department of Chemistry, Leuven, Belgium

## Abstract

The editorial introduces the Special Section on Molecular Neurophotonics.

Over the past 25 years, the development of new and improved molecular photonic tools has been pushing the frontiers of neuroscience ever forward. Representative examples of such tools include monomeric green and red fluorescent proteins, engineered luciferase–luciferin pairs, fluorescent and bioluminescent ${\mathrm{Ca}}^{2+}$ indicators, voltage-sensitive dyes, hybrid systems, bio-compatible nanoparticles, and optogenetic tools such as channelrhodopsins. Equally important to the molecules themselves (the “wetware”) have been the innovations in the hardware for neurophotonic applications, including light sources, detectors, and microscopes, as well as the software, such as AI-guided image acquisition and processing.

This special section on molecular neurophotonics (in *Neurophotonics* Volume 11 Issue 2) highlights advances in the broad area of molecular photonic tools with proven or potential utility in neuroscience. Reviews by Townsend et al. (10.1117/1.NPh.11.2.024204) and Porta-de-la-Riva et al. (10.1117/1.NPh.11.2.024203) provide overviews of recent advances in bioluminescent probes for neurobiology and of the use of bioluminescence as a functional tool for visualizing and controlling neuronal activity, respectively. The articles by Song et al. (10.1117/1.NPh.11.2.024201) and Aggarwal et al. (10.1117/1.NPh.11.2.024207) describe new fluorescent neural activity indicators for membrane potential and calcium ion (${\mathrm{Ca}}^{2+}$), respectively, making use of the color spectrum from far-red to blue. The contributions by Stern et al. (10.1117/1.NPh.11.2.024202), Silvagnoli et al. (10.1117/1.NPh.11.2.024206), and Celinskis et al. (10.1117/1.NPh.11.2.024209) focus on hardware and software aspects. Stern et al. (issue cover) describe an automated algorithmic framework to detect seizure-related events using ${\mathrm{Ca}}^{2+}$ imaging. Silvagnoli et al. developed a low-cost method for optimizing imaging parameters for bioluminescent sensors, and Celinskis et al. offer solutions for imaging hardware that allows multi-site imaging in the brain and spinal cord. The articles by Slaviero et al. (10.1117/1.NPh.11.2.024208), Björefeldt et al. (10.1117/1.NPh.11.2.021005), and Klein et al. (10.1117/1.NPh.11.2.024210) deal with bioluminescence-based optogenetics tools, specifically with luminopsins (LMOs), fusions of light emitting luciferases with light sensing optogenetic elements. Slaviero et al. systematically tested which design features in luciferase-opsin fusions contribute to the efficacy of LMOs, while Björefeldt et al. detailed the evolution and characterization of LMOs using novel luciferases optimized for Förster resonance energy transfer (FRET) when fused to a fluorescent protein. In an application study, Klein et al. describe advantages of LMOs for testing the role of choroid plexus dynamics in biological processes.

We hope that this collection of articles serves as a teaser to broaden interest in the rapidly evolving field of photonic tools and their many potential applications in basic and preclinical neuroscience research.

